# Case Report: Late presentation of post-coronary artery bypass surgery pericardial effusion heralding mediastinitis with tracheocutaneous fistula

**DOI:** 10.3389/fcvm.2025.1483395

**Published:** 2025-02-13

**Authors:** Muhammad Idu, Chiw Yeh Lim, Kim Chai Chua

**Affiliations:** ^1^ Department of Cardiology, National Heart Centre Singapore, Singapore, Singapore; ^2^ Department of Cardiothoracic Surgery, National Heart Centre Singapore, Singapore, Singapore

**Keywords:** case report, post-CABG pericardial effusion, mediastinitis, tracheocutaneous fistula, deep sternal wound infection (DSWI)

## Abstract

Post-coronary artery bypass surgery pericardial effusions are typically self-limiting but may rarely be significant, causing pericardial tamponade. We describe a case of late post-operative pericardial effusion that required pericardiocentesis and subsequent readmission for mediastinitis with the added complication of a tracheocutaneous fistula. Our case report is the first reported instance of pericardial tamponade heralding the onset of mediastinitis. It also describes the rare complication of tracheocutaneous fistula associated with mediastinitis.

## Introduction

Post-operative pericardial effusion after cardiac surgery is a relatively common finding, occurring in up to 64% of 780 patients undergoing cardiac surgery in a study by Pepi et al. (1). The majority of cases are asymptomatic and resolve spontaneously. However, up to 1.9% of patients develop features of cardiac tamponade, which occurs more often following valve surgery than coronary artery bypass surgery alone. Another study by Ashikhmina et al., involving 21,416 patients after cardiac surgery, detected symptomatic pericardial effusion in 1.5% of patients (2).

Post-operative pericardial effusion can be classified based on the timing of its development as early vs. late presentation (3). Early presentation pericardial effusion is defined as occurring within the first week of operation and is predominantly related to procedural-related bleeding. Late presentation pericardial effusion occurs after post-operative day 7 and is predominantly inflammatory-mediated. If symptomatic, patients may present with either features of pericarditis, such as pleuritic chest discomfort, or even features of pericardial tamponade, such as tachycardia, breathlessness, and low blood pressure. Patients who present with features of clinical pericardial tamponade would need to be treated with pericardiocentesis, which provides immediate relief of symptoms and hemodynamic stabilization.

Mediastinitis is an infrequent but serious complication that may manifest in the post-operative recovery period. Early detection and treatment can reduce morbidity. Our patient presented with late post-operative pericardial tamponade and improved clinically after pericardiocentesis. During his hospitalization, he did not exhibit any symptoms worrisome for mediastinitis, but unfortunately, he was later readmitted for this complication.

## Case description

Our patient is a 62-year-old man with cardiac risk factors of cigarette smoking, hypertension, and hyperlipidemia. He presented with unstable angina and was treated with an early elective coronary artery bypass surgery. His echocardiogram was normal, with a left ventricular ejection fraction of 60% and no pericardial effusion.

## Readmission for pericardial tamponade

He was readmitted 11 days later due to breathlessness, orthopnea, and mild pedal edema. He was afebrile, with a blood pressure of 118/79 mmHg and a heart rate of 102 beats per minute. Physical examination revealed muffled heart sounds and mildly elevated jugular venous pulsation. His chest x-ray showed a globular heart with intact sternal wires. Echocardiography showed a large pericardial effusion (measuring 4.1 cm) with features of early cardiac tamponade. His blood results were as follows: hemoglobin (Hb) 11.7 g/dl, white blood cell count (WBC) 13.8 × 10^9^/L, platelet count 397 × 10^9^/L, C-reactive protein (CRP) 13.8 mg/L, procalcitonin < 0.06 µg/L, and creatinine 103 µmol/L.

## Progress

**Figure 1:**
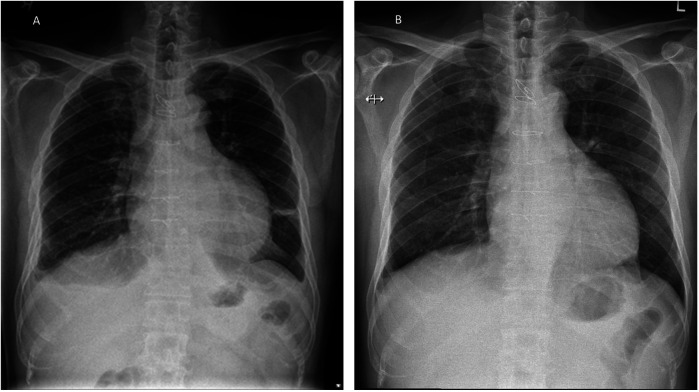
Patient's chest x-ray. **(A)** CXR during his admission for pericardial tamponade showing a globular heart and small pleural effusion. **(B)** Outpatient CXR taken 8 days later, showing that the third and fourth sternal wires had fractured with widening of the fifth and sixth wires.

He underwent urgent pericardiocentesis, during which 600 mL of hemoserous fluid was drained, resulting in the relief of symptoms. The pericardial drain was inserted in the cardiovascular suite under sterile conditions. No routine antibiotics were given. The pericardial drain was left *in situ* for 2 days and was removed once no further drainage was observed. Microscopic analysis of the fluid drained showed a WBC count of 473/µL, with 75% lymphocytes and 17% polymorphs. Gram staining did not identify any organisms. Aerobic, anaerobic, and acid-fast bacilli cultures were also negative. Fluid analysis showed a fluid lactate dehydrogenase (LDH) level of 384 (serum 408) U/L and a fluid protein level of 57 (serum 76) g/L, fulfilling Light's criteria^1^ for an inflammatory effusion. The cytology report showed occasional reactive mesothelial cells admixed with scattered small lymphocytes, some macrophages, and neutrophils in a hemorrhagic background, with no malignant cells identified. The patient remained clinically stable and was discharged on day 3 of admission. He was reviewed at the clinic 5 days later, complaining of a cough with purulent sputum. He was afebrile, and the previous sternotomy and pericardiocentesis sites were clean. A chest x-ray was done, which showed fractured sternal wires (Figure 1).

## Second readmission

**Figure 2:**
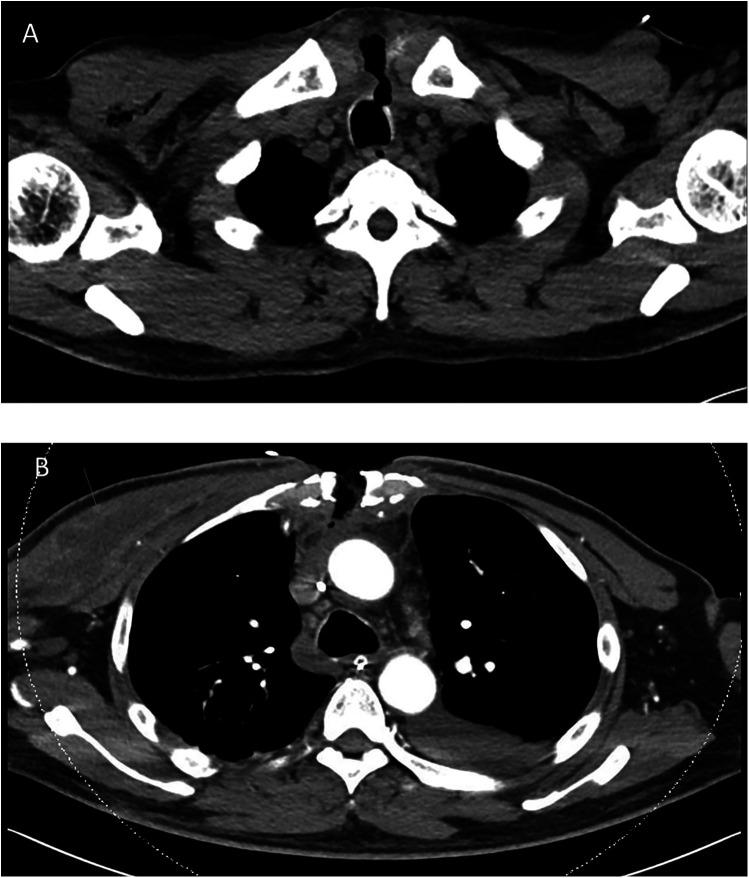
Patient's chest CT. **(A)** chest CT performed during his second readmission showing a tracheocutaneous fistula from the upper trachea to the subcutaneous sternum along the sternotomy defect. There was subcutaneous emphysema along the manubrium sternum, posterior sternum, and mediastinum, extending over the right chest wall. No large mediastinal collection was noted in the study. **(B)** Follow-up chest CT performed 1 week later showing interval development of a pectoralis major abscess (arrow), which required further surgical drainage.

The patient was readmitted 2 days later with complaints of worsening cough, blood-tinged sputum, orthopnea, pedal edema, fatigue, and purulent discharge from the upper part of his sternal wound. He was afebrile, with a blood pressure of 84/59 mmHg and a heart rate of 70 beats per minute. Physical examination revealed a small (3 mm) wound over his midline sternotomy that moved with respiration. His heart sounds were normal, but pulmonary crepitations were noted. A bedside echocardiogram showed a moderate-sized pericardial effusion (1.6 cm) without any features of tamponade. Blood test results were as follows: Hb 10.4 g/dL, WBC 14.5 × 10^9^/L, platelet count 99 × 10^9^/L, CRP 313 mg/L, procalcitonin 2.6 µg/L, and creatinine 314 µmol/L. An urgent chest CT showed a tracheal fistula to the subcutaneous sternum along the sternotomy defect and subcutaneous emphysema along the manubrium sternum, posterior sternum, and mediastinum, extending over the right chest wall. A small pericardial effusion and mid- and lower-sternal wire dehiscence were noted. The CT features were suggestive of mediastinitis, no large mediastinal collections were seen (Figure 2).

## Further management

**Figure 3:**
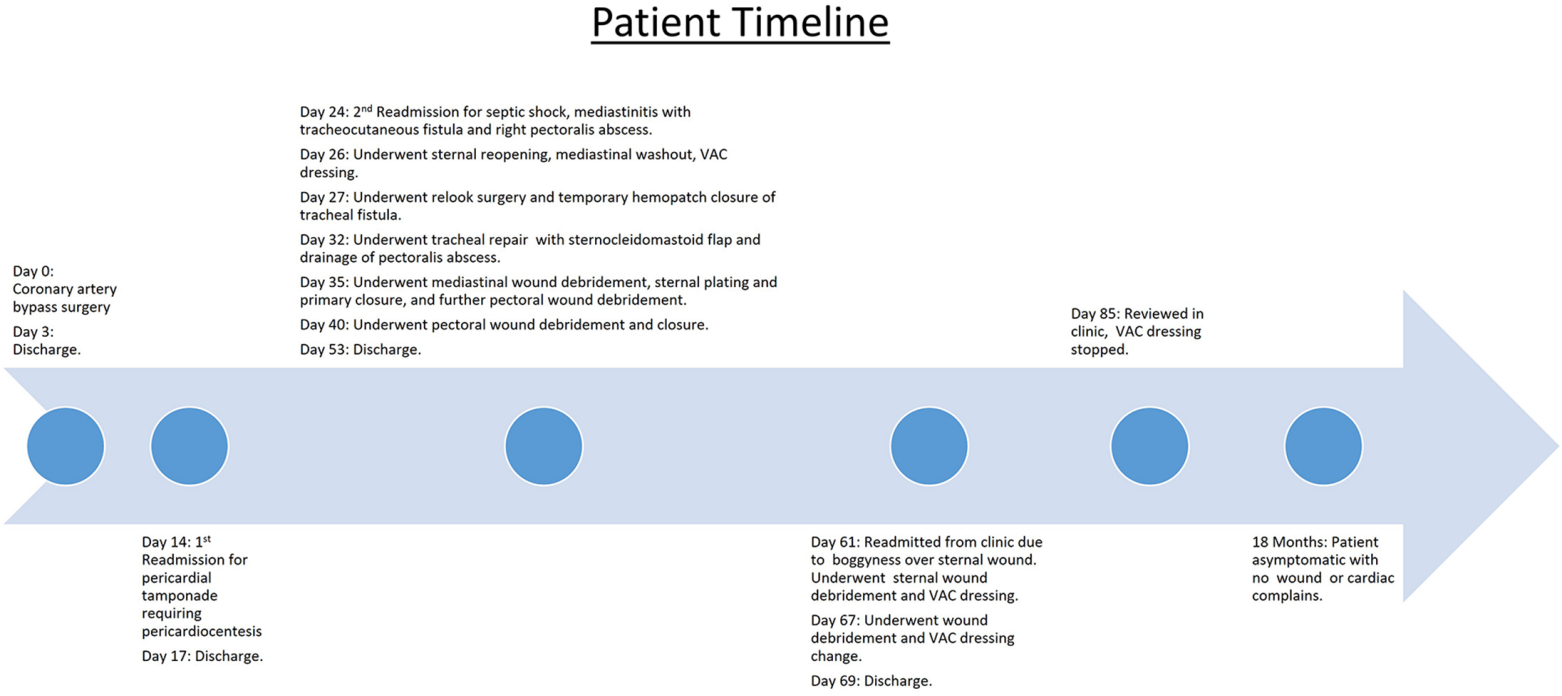
Timeline of events.

The patient was stabilized and underwent sternal reopening, mediastinal washout, and application of a vacuum-assisted closure (VAC) dressing. Intraoperatively, the lower four sternal wires were noted to be fractured, and unhealthy tissue with areas of fat necrosis was noted over the sternum. However, no frank pus or mediastinal abscess was observed. The following day, a relook surgery was performed, and a temporary seal of the tracheal defect was achieved with a Hemopatch. An interval chest CT at 1 week showed the development of an abscess in the right pectoralis major muscle. He underwent further surgery with an exploration of the neck, repair of the tracheal defect with a sternocleidomastoid muscle flap, debridement of the chest wound, and drainage of the right pectoralis major abscess. He eventually underwent sternal plating and primary closure of the right chest wall wound on post-operative day 9 from the sternal reopening surgery. Wound and sternal bone cultures grew *Klebsiella* and *Streptococcus anginosus*, while blood cultures grew *S. anginosus*. The patient was initially treated with intravenous meropenem and vancomycin before eventually switching to ceftriaxone and metronidazole. He was eventually discharged on post-operative day 17 with oral levofloxacin and metronidazole for an additional 4 weeks. One week after discharge, he was reviewed in the clinic and was noted to have a small area of skin erythema and bogginess (1.5 cm) over the upper part of his chest wound. He was readmitted for sternal wound debridement to the level of the sternal plate, followed by VAC dressing application until wound coverage was achieved 2 weeks later. He remained well during subsequent follow-up visits, and there were no further issues with wound healing or cardiac symptoms (Figure 3).

## Discussion

Mediastinitis is a feared complication of coronary artery bypass surgery with significant morbidity and mortality risk. Its incidence is low, with estimates ranging from 0.4% to 4%. In a study by Risenes et al. (4), the incidence of mediastinitis in a cohort of 18,532 patients was 0.6%, with a median presentation of 12 days (range 9–19 days) post-operatively. Risk factors include being a male patient, aged over 70 years, obesity (BMI ≥ 30), having received over 10 units of transfusions, diabetes, and chronic obstructive pulmonary disease (COPD). Mediastinitis is associated with significant morbidity, such as prolonged hospital stays, arrhythmia, stroke, and myocardial infarction. In addition, it is associated with reduced long-term survival at 10.3-years (49.5% vs. 71.0% survival). In a study by Milano et al. (5), out of 6,459 patients who underwent coronary artery bypass surgery, 1.3% developed mediastinitis. The 90-day mortality rate for the mediastinitis group was 11.8% vs. 5.5% for those without mediastinitis. An increase in interval mortality between 1 and 2 years post-surgery was also noted, with a rate of 8.1% in the mediastinitis group vs. 2.3% in the non-mediastinitis group.

The cause of post-operative mediastinitis is generally presumed to be either the intraoperative introduction of infection into the mediastinum or the spread of infection from the surgical wounds to the mediastinum (6). However, our patient demonstrated several atypical features. He presented with late pericardial effusion and features of pericardial tamponade. He was clinically not septic and showed resolution of symptoms after pericardiocentesis. Furthermore, his pericardial fluid cultures were negative for bacterial growth, and initial blood investigations did not suggest any signs of an ominous infection. Nevertheless, his white blood cell count and C-reactive protein levels were slightly elevated. Had we monitored these daily, they might have indicated that an infection was brewing, triggering an earlier chest CT. The follow-up chest x—ray, performed 2 days before his second readmission, also showed sternal wire fractures. Sternal wire fractures may be seen before mediastinitis manifests clinically and should prompt further investigations.

Tracheomediastinal fistulas are rare and more often seen in patients with cancer (7, 8) or following chemoradiation therapy (9). They have also been described after transbronchial biopsies (10). Routine airway intubation and ventilation have not been shown to cause tracheomediastinal fistulas. The tracheal fistula in our patient was likely from mediastinitis, with tracking of the infection to the trachea and the skin. Although no frank mediastinal abscess was noted on CT scan or intraoperatively, it is possible that an early mediastinal abscess could have drained via the fistula, both into the trachea (accounting for his cough) and subcutaneously to the skin. The infection likely also tracked subcutaneously to the right pectoralis major, where it later formed an abscess collection. In our patient, the most likely cause of his mediastinitis is a post-operative deep sternal wound infection; a less likely differential is bacterial seeding from the pericardiocentesis procedure. The deep sternal wound infection likely caused the mediastinitis and the reactive pericardial effusion.

Due to the significant morbidity and mortality associated with post-operative mediastinitis, delays in diagnosis and treatment should be avoided. As illustrated in this case report, patients may not clinically appear to be septic early on before overt mediastinitis manifests. In the setting of post-operative symptomatic pericardial effusion, a low threshold for CT imaging should be considered, especially if white blood cell count or inflammatory markers remain elevated.

## Conclusion

Post-coronary artery bypass surgery pericardial effusions are common and largely asymptomatic. A small minority of patients who present with pericardial tamponade may require pericardiocentesis. While most late-presentation pericardial effusions are reactive and self-resolving, they may be an early manifestation of mediastinitis. Mediastinitis is a major complication associated with significant morbidity and mortality and may present within the first 4 weeks post-operatively. Some patients may initially appear clinically well but may deteriorate rapidly. A high index of suspicion and a low threshold for chest CT should be considered, especially if inflammatory markers are persistently elevated or if sternal wire fractures are noted. Mediastinitis can lead to the rare complication of tracheal fistula.

## Data Availability

The original contributions presented in the study are included in the article/Supplementary Material, further inquiries can be directed to the corresponding author.

## Footnotes

^1^Light’s criteria suggest a fluid is exudative if fluid protein/serum protein ratio >0.5, fluid LDH/serum LDH ratio >0.6, or fluid LDH >2/3 of the upper reference range of serum LDH.

## Supplementary material

Supplementary Video 1 Video clip of Chest CT.
